# The Genotypic Characterization of *Cronobacter* spp. Isolated in China

**DOI:** 10.1371/journal.pone.0102179

**Published:** 2014-07-16

**Authors:** Jinghua Cui, Xiaoli Du, Hui Liu, Guangchun Hu, Guoping Lv, Baohong Xu, Xiaorong Yang, Wei Li, Zhigang Cui

**Affiliations:** 1 State Key Laboratory for Infectious Disease Prevention and Control, National Institute for Communicable Disease Control and Prevention, Chinese Center for Disease Control and Prevention, Beijing, China; 2 Jinan Municipal Center for Disease Control and Prevention, Jinan, China; 3 Shijiazhuang Municipal Center for Disease Control and Prevention, Shijiazhuang, China; 4 Sichuan Province Center for Disease Control and Prevention, Chengdu, China; Rockefeller University, United States of America

## Abstract

*Cronobacter* spp. (*Enterobacter sakazakii*) is an important pathogen contaminating powdered infant formula (PIF). To describe the genotypic diversity of *Cronobacter* isolated in China, we identified the isolates using *fusA* allele sequencing, and subtyped all of the isolates using pulsed-field gel electrophoresis (PFGE), multi-locus sequence typing (MLST), and multiple-locus variable-number tandem-repeat analysis (MLVA). A total of 105 isolates were identified, which included *C. sakazakii* (58 isolates), *C. malonaticus* (30 isolates), *C. dublinensis* (11 isolates), *C. turicensis* (5 isolates), and *C. muytjensii* (1 isolate). These isolates were showed to have 85 PFGE-patterns, 71 sequence types (STs), and 55 MLVA-patterns. Comparisons among the three molecular subtyping methods revealed that the PFGE method was the most distinguishable tool in identifying clusters of *Cronobacter* spp. through DNA fingerprinting, and MLST method came second. However, ESTR-1, ESTR-2, ESTR-3, and ESTR-4 were not effective loci for subtyping *Cronobacter* spp. such that the MLVA method requires further improvement.

## Introduction

Infants fed with powdered infant formula (PIF) contaminated with *Cronobacter* (*Enterobacter sakazakii*) can suffer from necrotising enterocolitis, bacteraemia, and neonatal meningitis [1]–[4]. Although these infections are relatively rare overall, they are of considerable significance, because infants comprise the highest-risk age group. While these infections are lethal for infants, recent studies reported possible diarrhea, wound infections, and urinary tract infections among immunocompromised people, particularly the elderly [5]–[8]. While the reservoir of *Cronobacter* is unknown in many cases, PIF was reported as a key source for infantile infection. Since 2004, the Food and Agriculture Organization of the United Nations (FAO) and the World Health Organization (WHO) have addressed the issue of *E. sakazakii* in PIF: accordingly, many studies of the phenotype and genotype of *Cronobacter* were conducted. In particular, *Cronobacter* was proposed as a new genus for *E. sakazakii*, and the genus consists of ten genomospecies [9]–[11]. These bacteria are distributed throughout nature and are found in plants, spice, herbs, water, dry foods, and starches [12]–[15]. Although there were no clinical infections reported, these strains have been widely isolated from food and from the environment in China.

Several molecular techniques have been developed for subtyping *Enterobacter sakazakii*, such as ribotyping, random amplification of polymorphic DNA, pulsed-field gel electrophoresis (PFGE), multiple-locus variable-number tandem-repeat analysis (MLVA), and multi-locus sequence typing (MLST) [16]–[18]. PulseNet International, which is the international molecular subtyping network for foodborne disease surveillance, considers PFGE as a gold-standard method for bacterial molecular typing. MLVA is the second generation of molecular subtyping method. MLST data is comparable across laboratories worldwide via the central curated database(http://pubmlst.org/cronobacter). To compare the genotypes of *Cronobacter* isolates from different sources, years and regions in China, we identified 105 *Cronobacter* isolates obtained from 2006 to 2011 from 11 Chinese provinces, and using PFGE, MLVA and MLST method to type these isolates. We also investigated which molecular subtyping method is more effective in determining the genetic relationship of *Cronobacter* isolates for the purpose of surveillance and outbreak investigation.

## Materials and Methods

### Bacterial strains

From 2006 to 2011,105 isolates were collected from 11 provinces in China, and their sources included infant food, potable water, and rectal swabs of healthy humans. Three rectal swabs from healthy humans were collected by Jinan municipal CDC, and the participants were informed and had provided their written consent to participate in this study, which was approved by the Jinan municipal CDC Ethics committee. As controls, the isolates ATCC 51329 and ATCC 29544 were provided by the Chinese Academy of Inspection and Quarantine (CAIQ). All isolates were confirmed by biochemical test using kit API 20E (bioMérieux, Marcy l′Etoile, France) and real-time PCR using a primer set and probe targeting the *dnaG* gene on the macromolecular synthesis operon [19]. Isolates were cultured on TSA agar (Oxoid, Basingstoke, United Kingdom), and they were cryopreservation at −80°C for long-term storage.

### PFGE

*Cronobacter* isolates were typed as described in the PulseNet standardised PFGE *Cronobacter* subtyping protocol [17]. *Xba*I and *Spe*I were the primary and secondary restriction enzymes, respectively. DNA fragments were separated by electrophoresis (CHEF Mapper, Bio-Rad Laboratories, Hercules, California, US) through a 1% (w/v) agarose gel (Seakem Gold, Rockland, Maine, US) in 0.5× TBE buffer at 6 volts/cm using an initial switch time of 1.8 s and a final switch time of 25 s. Gels were stained in deionised water containing GelRed Nucleic Acid Gel Stain (Biotium, CA, US), and visualised under U.V. light using a GelDoc XR^+^system (Bio-Rad laboratories, Hercules, California, US). *Xba*I-digested *Salmonella* serotype Braenderup H9812 was used as the molecular weight standard. Dendrograms were created using BioNumerics software version 5.1 (Applied Maths, Sint-Martens-Latem, Belgium) using the DICE coefficient, the unweighted pair group method with arithmetic means (UPGMA), and a band position tolerance of 1.5%.

### Genomic DNA Isolation

Bacteria were cultured on TSA agar at 37°C for 18–24 h, and an isolated colony was inoculated into 5 ml tryptone soy broth (Oxoid, Basingstoke, UK). Bacteria from 1 ml of overnight culture was recovered by centrifugation at 10,000×*g* for 2 min. Total DNA was prepared using the QIA amp DNA Mini kit (Qiagen, Maryland, US) and quantified using a spectrophotometer (IMPLEN, Gemany) for MLST and MLVA method.

### MLST

The seven multilocus loci of *Cronobacter* were *atpD*, *fusA*, *glnS*, *gltB*, *gyrB*, *infB*, and *ppsA*. The primers and the detailed amplification conditions for these fragments were available at http://pubmlst.org/cronobacter/info/protocol.shtml; Baldwin *et al*. 2009. The amplified fragments were purified using a PCR purification Kit (Qiagen, Maryland, US) according to the manufacture's instructions. DNA sequencing was performed with an ABI 3730XL DNA Analyzer (Applied Biosystems, CA, US). In order to identify *Cronobacter* species, the phylogenetic and molecular evolutionary analyses of *fusA* allele were conducted by using MEGA (Molecular Evolutionary Genetics Analysis) version 5.0 [20]. Concatenated DNA sequences for sequence types not detected in the present study were downloaded from the MLST database.

### MLVA

Four loci, ESTR-1, ESTR-2, ESTR-3, ESTR-4 [16] were amplified using the primers at a concentration of 0.2 uM with EX Taq DNA polymerase (TBI, Dalian, China) in a 20 µl final reaction volume using the manufacturer's reaction buffer. Following amplification, all PCR products were electrophoresed using an Applied Biosystems 3730XL DNA Analyser (Applied Biosystems, CA, US). The band intensity of each amplicon was examined prior to analysis with Gene Mapper 4.0 software (Applied Biosystems, CA, US). All four loci were amplified three times from each isolate to ensure reproducibility. To confirm the numbers of alleles at the four loci, PCR products were processed using the BigDye kit and sequenced on an ABI 3730XL capillary DNA analyser (Applied Biosystems, CA, US).

## Results

### Species identification

**Figure 1:**
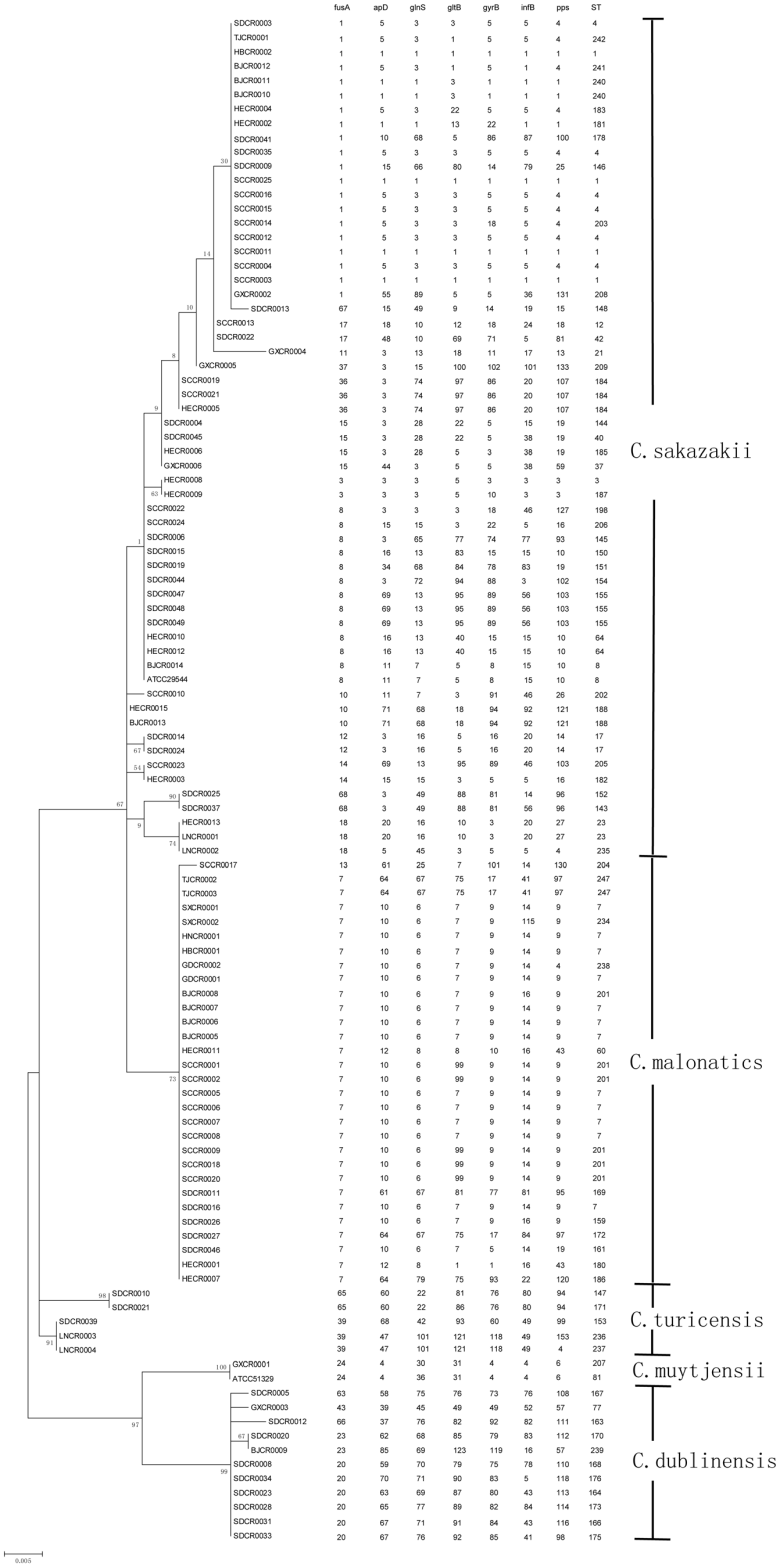
Maximum likelihood tree of the *fusA* alleles and MLST profiles for the differentiation of *Cronobacter* species in this study.

Based on Joseph's study [18], the 105 isolates in this study were identified as follows: *C. sakazakii* (58 isolates), *C. malonaticus* (30 isolates), *C. dublinensis* (11 isolates), *C. turicensis* (5 isolates), and *C. muytjensii* (1 isolate) (Fig. 1). In addtion, the complete 16S rRNA gene was also sequenced and additional biochemical tests were performed, including the indole test, acid production from dulcitol and methyl-a-D-glucopyranoside, and malonate utilization (data not showed).

### PFGE

**Figure 2:**
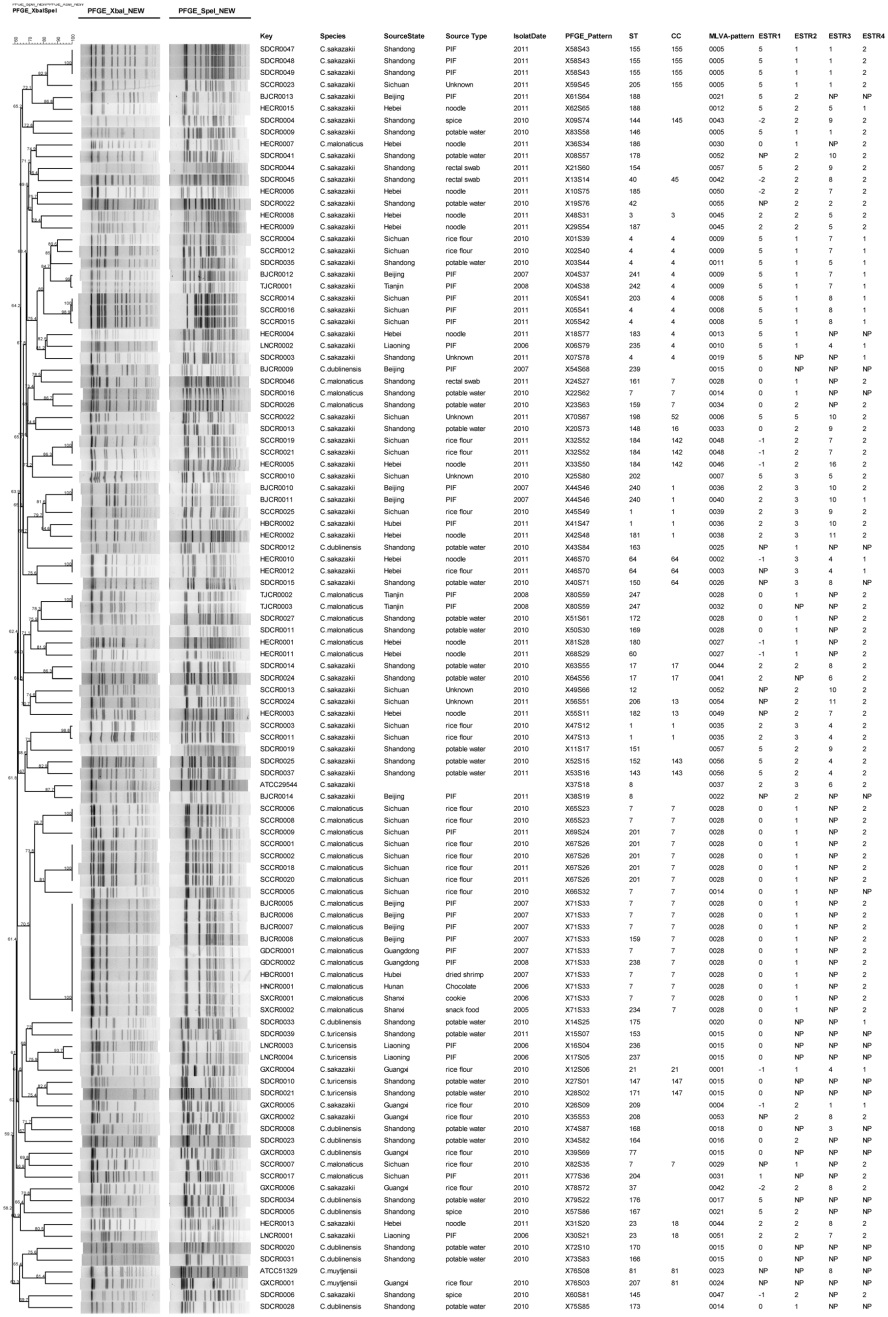
Dendrogram combining PFGE patterns of *Xba*I- and *Spe*I- digested DNA from *Cronobacter* spp. in this study. Isolates information, STs, CC and MLVA allele string are also included.

BioNumerics software analysis showed the 105 isolates demonstrated 85 distinguishable *Xba*I-*Spe*I PFGE patterns, and the control strains (*C. sakazakii* ATCC29544, *C. muytjensii* ATCC51329) showed the two PFGE patterns different from the isolates in this study (Fig. 2). A high degree of genetic diversity was revealed using PFGE after genomic DNA digestion, and the discriminatory index was 0.9940, as it was calculated using Simpson's diversity index. Nevertheless, when the isolates digested with *Xba*I and *Spe*I respectively, 83 and 86 PFGE patterns were demonstrated. So the identification rate of PFGE with *Spe*I may be higher than with *Xba*I.

### MLST

The seven defined alleles of each isolate were sequenced, and each house keeping gene *atpD*, *fusA*, *glnS*, *gltB*, *gyrB*, *infB*, and *ppsA* was found to possess 14, 5, 15, 25, 24, 14, and 27 new types of allele sequences, respectively. All the sequences were submitted to the MLST database and assigned new numbers, which were showed in Fig. 1. The 105 strains were in 71 STs, of which only 15 types had been reported by other countries. Although many new STs were found, these STs belonged to previous clonal complex (CC, single or double locus variant of dominant sequence type), such as ST183, 203, 235, 241, 242 belonged to ST4; and ST159, 161, 201, 238 belonged to ST7; and so on. The isolate *C. sakazakii* ATCC29544 showed the same ST with one isolate (BJCR0014), whereas *C. muytjensii* ATCC51329 showed different ST from all the isolates in this study. Collectively, the above results suggest *Cronobacter* isolated from China had a high diversity, particularly for the isolates identified to *C. dublinensis* and *C. turicensis*.

### MLVA

**Figure 3:**
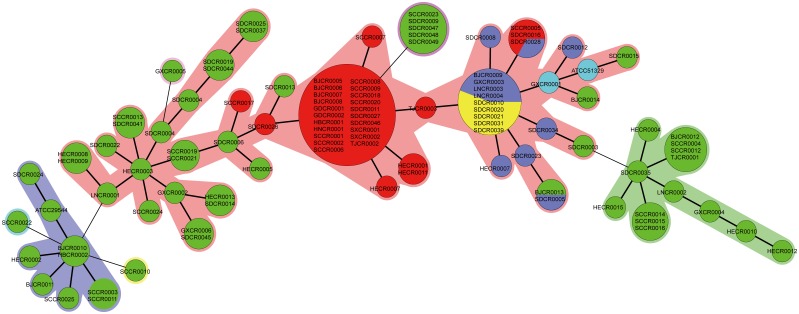
MST of MLVA analysis of 107 isolates of *Cronobacter* spp. in this study. Different species were labelled with different colour: *C. sakazakii* with green, *C. malonaticus* with red, *C. dublinensis* with purple, *C. turicensis* with yellow, and *C. muytjensii* with blue.

Using the MLVA method developed by Mullane *et al.*, we calculated the numbers of alleles for the ESTR-1, ESTR-2, ESTR-3, and ESTR-4 loci: they were 6, 4, 12, and 2, respectively. For certain isolates, there was no PCR amplicon at a variable number of tandem repeats (VNTR) locus, which was labeled as “NP” meaning“no product”, and the amplicon was absent in 11% of the isolates at the ESTR-1 locus, 17.1% at the ESTR-2 locus, 49% at the ESTR-3 locus, and 22% at the ESTR-4 locus. The genetic diversity of VNTRs for each locus was calculated using Simpson's diversity index, and the values for ESTR-1, ESTR-2, ESTR-3, and ESTR-4 loci were 71.68%, 61.16%, 89.55%, and 32.67%, respectively. Although the polymorphism indicator at the ESTR-3 locus was the best, the rate of missing amplicons was very high. For VNTRs in ESTR-1 locus, the upstream and downstream sequences were not conserved in 12% of the isolates. To distinguish these results from conserved sequences, the repeat copy number was determined by negative correlation (Fig. 2). Minimum spanning tree (MST) revealed that the 105 isolates were divided into 55 MLVA patterns, and the control strains (ATCC29544, ATCC51329) showed two MLVA patterns different from the isolates in this study. The isolates belonging to the different species were gathered in one cluster (Fig. 3)

## Discussion

Iversen *et al*. used f-AFLP fingerprints, ribopatterns, full-length 16S rRNA gene sequences, and biochemical tests for species typing in previous study [9]. While 16S rRNA gene sequences failed to distinguish *C. sakazakii* from *C. malonaticus*, and lack of continuity between *Cronobacter* biotypes and species was reported [21]. Here the isolates' species identification was determined by *fusA* allele sequence typing based on Joseph's study. Similar to other studies [22], [23], *C. sakazakii* was isolated from all sources, and was the most common *Cronobacter* species. Except for four isolates from food sources, the other 12 isolates, which were classified as *C. dublinensis* or *C. turicensis*, were from environment. This indicats that different species of *Cronobacter* is prevalent in different econiche. In addition, of the three isolates from rectal swabs of healthy individuals, two were identified as *C. sakazakii* (ST40, CC45; ST154), and one was identified as *C. malonaticus* (ST161, CC7). It has been reported that the *Cronobacter* spp. that cause disease were mainly *C. sakazaikii* and *C. malonaticus*. These results support the hypothesis that *C. sakazakii* and *C. malonaticus* may be associated with humans while other species with plants and the environment [24].

PFGE is well-established and widely used for the molecular subtyping of bacteria, including *Enterobacter sakazakii* [25]–[28]. Brengi *et al.* modified the PulseNet standard protocol in 2012, and we performed and evaluated its applicability for analyzing the isolates from China. Most isolates with the same *Xba*I-*Spe*I PFGE pattern were isolated from the same source, region and year, except for one cluster (PFGE-pattern X71S33). The strains of this cluster were from 5 provinces, and isolated from 2005 to 2008, which maybe demonstrate this type of isolates was prevalent at that time in China. We also found that the cluster (PFGE-pattern X67S26) was existent in Sichuan Province from 2010 to 2011. Unexpectedly, the fingerprint similarity rate from the same species was not high. Although several isolates demonstrated the same PFGE pattern when they were digested with *Xba*I, they showed different PFGE patterns when using *Spe*I, and the converse was true. Therefore, it was important to perform PFGE on *Cronbacter* using at least two restriction enzymes as a useful tool for surveillance and outbreak investigation.

The *fusA* gene was more conservative comparing with other house-keeping genes, only five new alleles were found in 105 isolates, so it was more suitable for species. While, other genes possessed more than ten types of new alleles, with the result that many novel STs, which were quite different from the alleles reported by other countries, were found in this study. In the same STs, some parts of isolates from the same region and time, such as ST155, ST17, ST201, ST 240, and ST247; however, the isolates of ST7, ST4, ST1, ST23, ST184, and ST188 were from different source, region and time. MLST method was not enough distinguishable for subtyping *Cronobacter* spp., but it was a convenient method to compare the data across laboratories worldwide. For example, *C. sakazakii* CC4 was reported to be associated with neonate meningitis by other countries [29], while it was found in food and water in China. There were 3 isolates from healthy individuals in China were identified to *C. sakazakii* ST40 (CC45), *C. sakazakii* ST154, and *C. malonaticus* ST161(CC7), which have not been reported to associated with clinical cases.

MLVA-Pattern 28 had the most number of isolates, while the isolates from this cluster were from the different regions, sources, and year. The identification rate of MLVA method was not high. Mullane *et al.* obtained similar results in their study, and they attributed this to the *C. sakazakii* strain ATCC BAA-894 VNTR-based primers, which were designed prior to the *Cronobacter* genus definition. In our study, it was found that the isolates that failed to amplify did not always belong to *C. sakazakii*. Therefore, this MLVA method was limited to the full genus as currently recognized, and should be further optimized for effective subtyping of *Cronobacter*.

Comparing the three molecular subtyping methods by the Simpson's diversity indexes, the D-value of PFGE method was the highest for each species (Table 1). Although certain isolates with the same PFGE-pattern were not in same STs, they were in same CCs, while the opposite results were not obtained; certain isolates with similar PFGE-Patterns were not gathered in one cluster using MLVA methods: the opposite results also were obtained.

**Table 1:** Numbers of patterns and D-values of PFGE, MLST and MLVA among different *Cronobacter species.*

	PFGE		MLST		MLVA	
	No. of patterns	D	No. of patterns	D	No. of patterns	D
*C. sakazakii* (n = 58)	52	0.9958	41	0.9806	40	0.9831
*C. malonaticus* (n = 30)	16	0.8782	13	0.8207	8	0.5126
*C. dublinensis* (n = 11)	11	1.0000	11	1.0000	8	0.8909
*C. turicensis* (n = 5)	5	1.0000	5	1.0000	1	0.0000
*C. muytjensii* (n = 1)	-	-	-	-	-	-

## Conclusions

In conclusion, there were polymorphisms between the *Cronobacter* spp. isolated in China. The PFGE method was a useful tool in identifying clusters of *Cronobacter* spp. through DNA fingerprinting, and assisted in retrospective investigations to identify food and environmental sources of *Cronobacter*. Although the D-value of the MLST method was lower than the PFGE method for each species, it was a convenient method to compare the data across laboratories worldwide. However, ESTR-1, ESTR-2, ESTR-3, and ESTR-4 were not effective loci for subtyping the *Cronobacter* spp. from China. And new loci are required to establish an MLVA-based protocol in the future.
